# Stroke as initial manifestation of non-small cell lung cancer with Trousseau syndrome

**DOI:** 10.1186/s12885-023-11627-2

**Published:** 2023-11-10

**Authors:** Weiping Hong, Tongtong Zhang, Changguo Shan, Hainan Li, Tao Lin, Da Liu, Junjie Zhen, Juan Li, Mingyao Lai, Zhaoming Zhou, Cheng Zhou, Meijuan Zhou, Minghua Wang, Linbo Cai, Lei Wen

**Affiliations:** 1 Department of Oncology, Guangdong Sanjiu Brain Hospital, 578 Shatai North Road, Guangdong, 510510 Guangzhou China; 2 Department of Nuclear Medicine, The First Affiliated Hospital of Guizhou Medical University, 58 Guiyi Street, Guizhou, 550000 Guiyang China; 3 Department of Pathology, Guangdong Sanjiu Brain Hospital, Guangzhou, China; 4 Department of Neurosurgery, Guangdong Sanjiu Brain Hospital, Guangzhou, China; 5 Department of Radiation Oncology, Nanfang Hospital, Southern Medical University, Guangzhou, China; 6 Department of Radiation Medicine, School of Public Health, Southern Medical University, Guangzhou, China; 7 Department of Radiation Oncology, Zhujiang Hospital, Southern Medical University, 253 Gongye Dadao, Guangdong, 510280 Guangzhou China

**Keywords:** Stroke, Lung Adenocarcinoma, Trousseau Syndrome

## Abstract

**Objective:**

Stroke is a rare but fatal complication of advanced cancer with Trousseau syndrome, especially as initial symptoms. Here, we report the clinical characteristics, treatment, and prognosis of patients with non-small cell lung cancer (NSCLC) who initially presenting with acute multiple cerebral infarction.

**Methods:**

The clinical characteristics, imaging, treatment, and oncological outcomes of 10 patients diagnosed with Trousseau syndrome and NSCLC between 2015 and 2021 at Guangdong Sanjiu Brain Hospital were retrospectively collected and analyzed. The clinical course of two typical cases were presented.

**Results:**

All 10 patients with pathologically confirmed lung adenocarcinoma initially presented with neurological symptoms, including hemiplegic paralysis (7 patients, 70%), dizziness (5 patients, 50%), and unclear speech (3 patients, 30%). The median age was 63.5 years. Eight and two cases were stage III and IV, respectively, at the initial diagnosis. Five patients underwent driver gene testing, revealing three patients with EGFR-sensitive mutations, one patient with ALK fusion, and one patient with wild-type EGFR. All 10 patients received antiplatelet therapy, and six patients subsequently received anti-cancer treatment. The median overall survival of the patients was 8.5 months (95% confidence interval) and 1-year survival rate was 57.1%. Patients who received antitumor treatment, especially those harboring driver gene mutations and received tyrosine kinase inhibitors, had better neurological symptom recovery and superior oncological prognosis (median overall survival, not reached versus 7.4 months, *p* = 0.038).

**Conclusion:**

Trousseau syndrome, presenting as multiple cerebral infarctions, is a rare complication of lung adenocarcinoma. Both antiplatelet and antitumor treatment are recommended to achieve better neurological recovery and oncological prognosis in these patients.

## Background

Lung cancer ranks first incidence among cancers and accounting for one-fifth of all cancer-related mortality worldwide [1]. Although advanced diagnostic and therapeutic strategies have been used in recent years, the prognosis of lung cancer remains unsatisfactory, partly associated with delayed diagnosis. The most common symptoms are respiratory-related symptoms, such as cough, sputum, hemoptysis, and chest tightness. However, arterial thromboembolism as the first presentationof lung cancer is not so common, especially stroke which is relatively rare as the initial symptom.

Cancer patients are in a hypercoagulable state and are at higher risk for the development of cancer-associated thromboembolism than those without cancer. Almantroso et al*.* first identified the relationship between thrombosis and cancer in 1865, and several studies have confirmed that thrombosis is a common pathogenic condition in patients with cancer [2]. Trousseau’s syndrome (cancer-associated thrombosis) is a common complication and the second leading cause of death in cancer patients [3]. The reasons of the abnormal coagulation function are associated with general patient-related risk factors, and other factors that are specific to the particular cancer type or treatment. The incidence of arterial thrombosis is estimated to be 2–5%, accounting for 10–30% of all thrombotic complications [4, 5]. Brain is injured by this pathophysiology because of the rich distribution of the procoagulation factor, thromboplastin, combined with the low levels of the anticoagulation factor, thrombomodulin, in the brain epithelium [6]. Among all cancers, lung cancer shows a higher incidence of thrombosis, followed by rectal and prostate cancer; however, lung cancer with stroke as the first symptom has rarely been reported.

In this study, we retrospectively reviewed the clinical data, treatment and prognosis of 10 patients with lung adenocarcinoma who presented with stroke as the first symptom.

## Methods

### Patients

Patients diagnosed with non-small cell lung cancer (NSCLC) and Trousseau syndrome from January 1, 2015, to December 31, 2021, at Guangdong Sanjiu Brain Hospital were screened. Patients were included if they met the following criteria: 1) initially presenting with acute neurological-related symptoms and signs, such as hemiplegia, dizziness, slurred speech; 2) imaging manifestation of acute ischemic infarction in multiple areas of the brain using magnetic resonance imaging (MRI), and the diagnosis of acute cerebral infarction were confirmed by an experienced neurologist and radiologist; 3) pathological diagnosis of lung cancer; 4) acute cerebral infarction and NSCLC were diagnosed simultaneously (within 1 month); 5) those with brain metastases, primary brain tumors, hematological system tumors, and other diseases that could induce acute cerebral infarction, such as atrial fibrillation, rheumatic heart disease, and heart failure, were excluded. This study was approved by the Ethics Committee of Guangdong Sanjiu Brain Hospital.

### Data collection

The demographic and clinicopathological data of all patients were collected, including age, gender, comorbidities (hypertension, diabetes), laboratory findings (D-dimer, serum carcinoembryonic antigen [CEA]), as well as histological classification of lung cancer. Lung cancer was staged according to American Joint Committee on Cancer (8th edition). The National Institutes of Health Stroke Scale (NIHSS) score index was used to measure the neurological impairments caused by cerebral infarction. Data of gene mutation status were collected when available. Treatment strategies, including antiplatelet and antitumor therapy (targeted therapy, chemotherapy, radiatherapy and surgery) were collected. The Response Evaluation Criteria In Solid Tumors (v1.1) was used to assess the efficiency of antitumor treatment.

### Statistical analysis

Continuous variables, such as age, serum CEA levels, and D-dimer levels were described as median (range). Categorical variables are described using absolute numbers and percentages. Overall survival was defined as the time from the diagnosis of NSCLC to death or the time to the last follow-up. Kaplan–Meier survival curves were generated and compared using log-rank tests. All statistical analyses were performed using the R software (https://cran.r-project.org/).

## Results

### Patient characteristics

Ten patients diagnosed with NSCLC and acute stroke met the inclusion criteria and were included in the present study. The median age was 63.5 years (range, 56–77 years); 6 patients were men and 4 were women. All patients initially presented with neurological symptoms, including hemiplegic paralysis (7 patients, 70%), dizziness (5 patients, 50%), and unclear speech (3 patients, 30%), etc. The median NIHSS score was 5 (range, 0–14) when admitted to hospital. All patients were pathologically diagnosed with primary lung adenocarcinoma within one month of the first presentation of ischemic stroke. Eight patients had stage III and two patients had stage IV disease at initial diagnosis. Among the 10 patients, six had a history of hypertension, three had a history of smoking, and two had diabetes mellitus and hyperlipidemia (Table 1).

**Table 1:** Clinical characteristics of 10 NSCLC patients initially presented with Trousseau Syndrome

Case	Age	Sex	NIHSS	KPS	Pathology	D-Dimer (mg/L)	CEA (ng/L)	BM	Medical history	Symptoms
1	56	M	5	30	AD	8.23	126	No	Hypertension, Smoking	Dizzy, unclear speech, facial paralysis, hemiplegic paralysis, mental disorder
2	63	F	8	50	AD	0.84	17.14	No	Hypertension, Hyperlipidemia, Diabetes	Hemiplegic paralysis, motor aphasia
3	56	M	11	50	AD	34.18	4.56	No	Smoking	Unclear speech, hemiplegic paralysis
4	58	F	14	60	AD	28.9	1.1	No	Hypertension, Hyperlipidemia, Diabetes	Hemiplegic paralysis, lethargy
5	63	F	1	80	AD	18.86	26.03	No	-	Hemiplegic paralysis, blurred vision
6	69	M	4	60	AD	7.24	11.3	No	Hypertension	Dizzy, unclear speech, facial paralysis
7	77	M	0	60	AD	3.04	4.31	No	-	Dizzy, blurred vision, nausea
8	66	M	14	20	AD	4.5	4.25	No	Smoking	Lethargy, hemiplegic paralysis
9	64	M	5	40	AD	0.31	125	No	-	Dizzy, hemiplegic paralysis, facial paralysis
10	71	F	0	50	AD	3.04	1.82	No	Hypertension	Dizzy, Vomiting

*Abbreviations*: *AD* Adenocarcinoma, *BM* Brain metastases, *CEA* Carcinoembryonic antigen, *NIHSS* National institute of health stroke scale, *KPS* Karnofsky performance status

### Laboratory, imaging and pathological findings

**Fig. 1:**
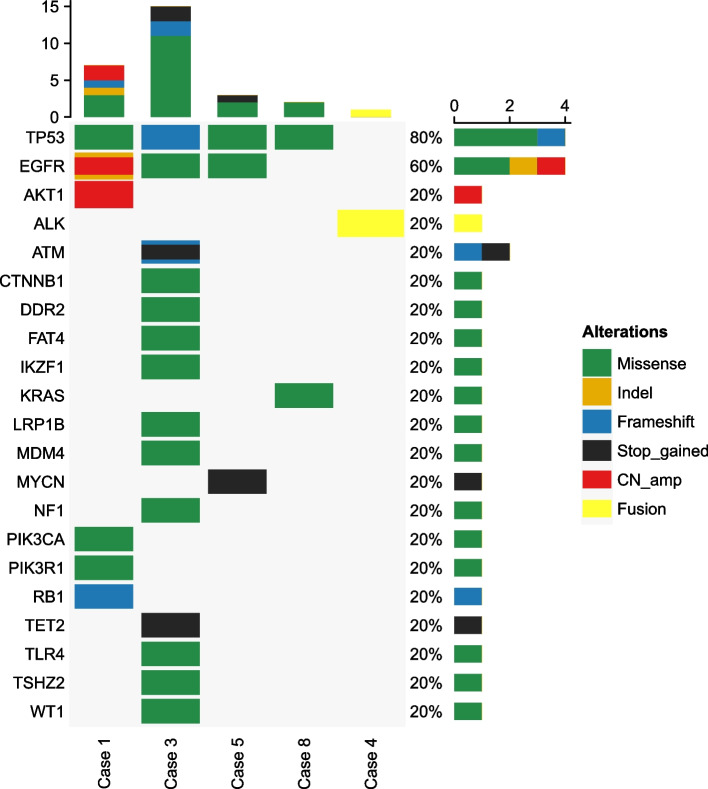
Heatmap for altered genes in five patients with lung cancer and cerebral infarction

D-dimer (median, 5.87 mg/L; range, 0.31–34.18 mg/L) were elevated in all 10 patients at initial diagnosis. Tumor marker CEA was elevated in five patients while it was detected in normal levels in the rest five patients (7.93 ng/L; range, 1.1–126 ng/L) (Table 1). Cranial MRI DWI revealed multifocal diffuse signal restriction, suggesting multiple acute cerebral infarction in all 10 patients. Enhanced MRI did not reveal obvious parenchymal or meningeal metastases. Pathological diagnosis (HE and immunohistochemistry) of all 10 patients supported lung adenocarcinoma. Five patients underwent gene analysis by using next-generation sequencing with a 168-gene panal from Burning Rock Biotech (Guangzhou, China) which was described in our previous study [7], identifying EGFR-sensitive mutations in three cases, ALK fusion in one case, and wild-type in one case (Fig. 1).

### Treatment and prognosis

**Fig. 2:**
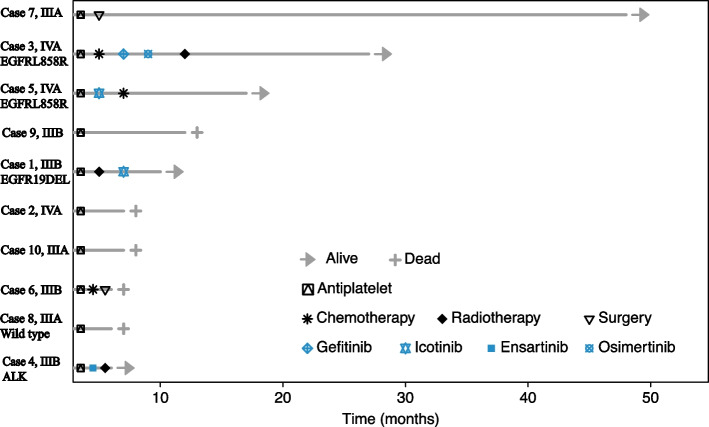
Treatment, response, and survival status of 10 patients by swimming plot

**Fig. 3:**
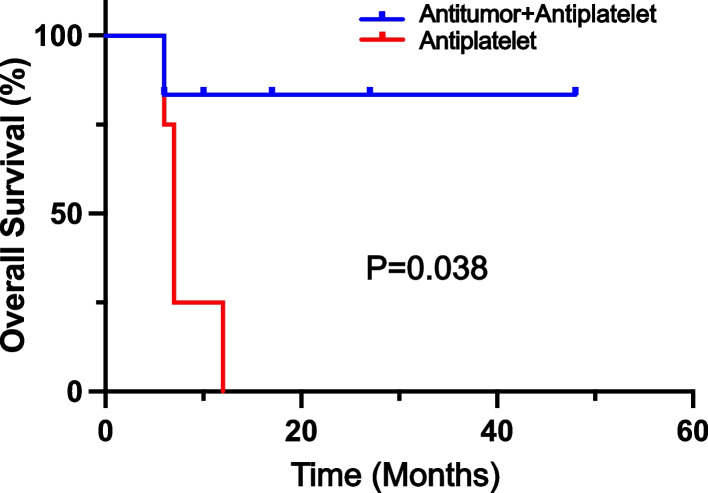
Kaplan–Meier estimates of overall survival according to treatment group. Median overall survival was not reached in anti-tumor plus anti-platelet group and 7.4 months in anti-platelet only group (*p* = 0.038)

All 10 patients received antiplatelet therapy (aspirin and/or clopidogrel) when an ischemic stroke was diagnosed. Six patients received further anticancer treatment after primary lung adenocarcinoma was confirmed. After a median follow-up of 17.9 months, five patients were dead and five were still alive (Fig. 2). The median overall survival were 12.1 months (95%CI: 1.1 to 23.1 months) and one year overall survival rate was 57.1%. First-generation EGFR tyrosine kinase inhibitors (TKI) were administered to three patients with EGFR mutations (icotinib in two patients and gefitinib in one patient) and partial response and stable disease were reached in 2 and 1 patients, respectively. One patient with ALK fusion was treated with ensartinib and persistent partial response was acheived. Two patients with stage IIIA disease underwent surgical resection, and one patient was dead and one was alive. Four patients did not receive any anticancer treatment for personal reasons and all of them were dead. (Table 2). The median overall survival was significantly longer in patients who received both anticancer and antiplatelet treatment than in those who received antiplatelet treatment only (not reached versus 7.4 months, *p* = 0.038) (Fig. 3).

**Table 2:** Treatment, response, and survival status of 10 patients

Case	Stage^a^	Gene Mutation	Anti-tumor	Anti-platelet	Tumor response	Neurological condition	Status		OS (mo.)
1	T3N2M0, IIIB	EGFR 19DEl	Icotinib; Lung radiotherapy	Aspirin, Clopidogrel	PR	Marked Improved	Alive	10	
2	T1N2M0, IIIA	NA	No treatment	Clopidogrel	NA	Stable	Dead	7	
3	T2N1M1, IVA	EGFR L858R	Gefitinib; Lung radiotherapy	Aspirin	SD	Stable	Alive	27	
4	T1N1M0, IIIB	ALK fusion	Ensartinib; Lung radiotherapy	Aspirin, Clopidogrel	PR	Marked Improved	Alive	6	
5	T1N3M1, IVB	EGFR L858R	Icotinib; Chemotherapy	Clopidogrel	PR	Marked Improved	Alive	17	
6	T2N3M0, IIIB	NA	No treatment	Clopidogrel	NA	Stable	Dead	6	
7	T1N2Mo, IIIA	NA	Surgery	Aspirin	NED	Improved	Alive	48	
8	T2N2M0, IIIA	Wild Type	Chemotherapy; Surgery	Aspirin, Clopidogrel	PD	Improved	Dead	6	
9	T2N1M0, IIIB	NA	No treatment	Aspirin, Clopidogrel	NA	Stable	Dead	12	
10	T2N2M0, IIIA	NA	No treatment	Clopidogrel	NA	Stable	Dead	7	

^a^According to the American Joint Committee on Cancer (8th edition)

*Abbreviations*: *OS* Overall survival, *EGFR* Epidermal growth factor receptor, *ALK* Anaplastic lymphoma kinase, *PR* Partial response, *SD* Stable, *PD* Progressive disease, *NED* No evidence of disease, *NA* Not available

### Typical cases

**Fig. 4:**
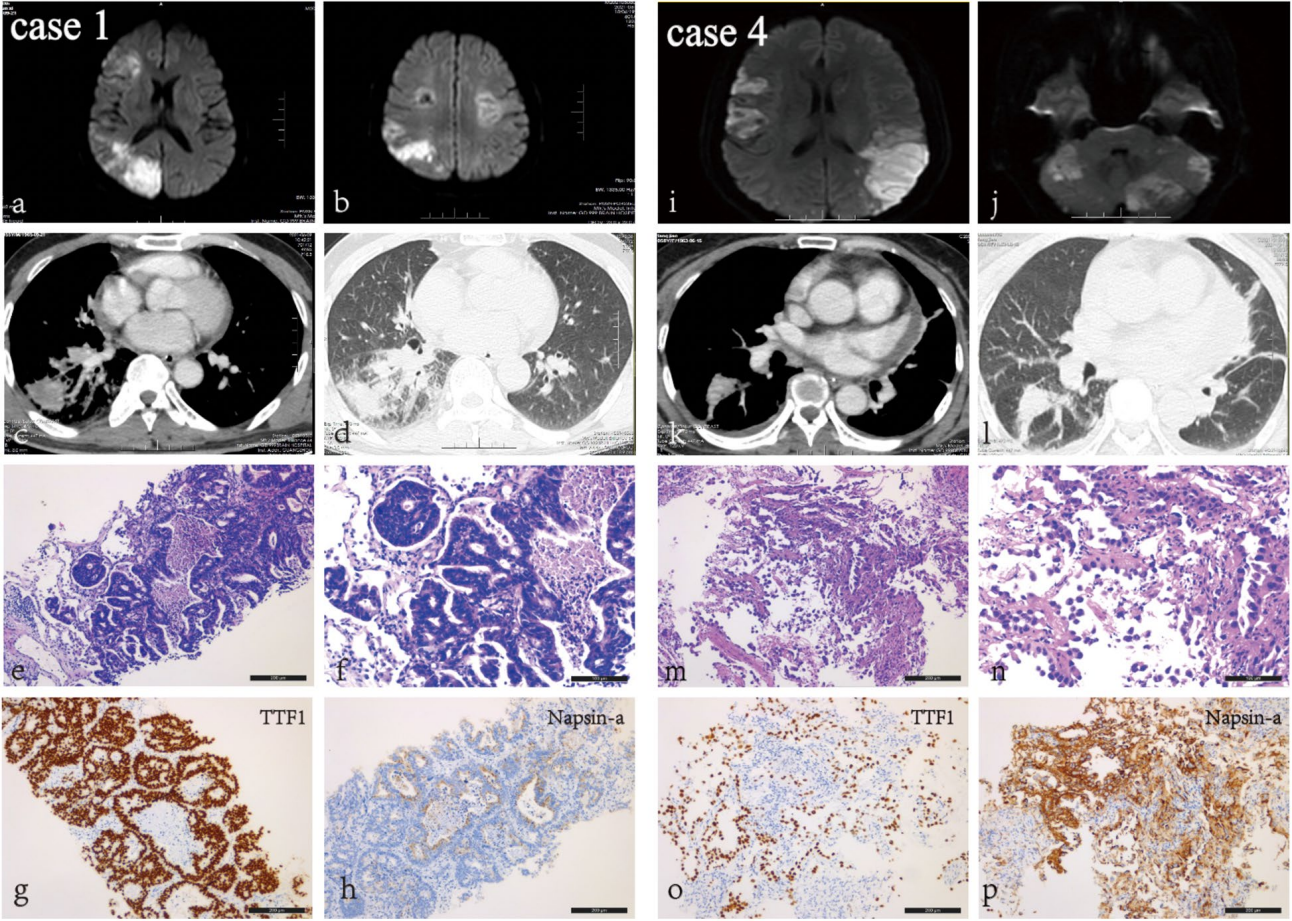
Imaging and pathological results of two representative patients diagnosed with primary lung adenocarcinoma and multiple cerebral infarctions. Both patients presented with stoke-related symptoms and brain MRI DWI revealed acute infarctions in multiple cerebrum (a, b, i, j). Lung mass was found by chest CT (c, d, k, l) and primary lung adenocarcinoma were confirmed pathologically (HE and immunohistochemistry) (e–h, m-p). (Black bars in Fig. 4f and 4n reprent 100um, black bars in Fig e, m, g-p represent 200um)

**Fig. 5:**
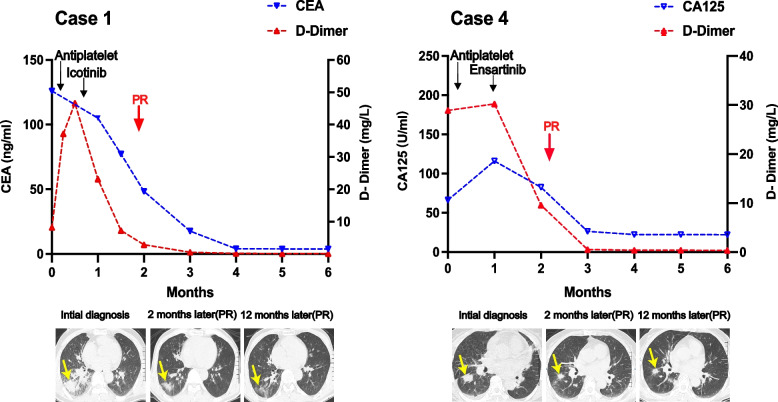
Dynamic serum CEA, D-dimer and radiological response changes in 2 representative patients

Case 1 was a 53-year-old man presented with dizzy, unclear speech, facial paralysis, hemiplegic paralysis and mental disorder. The NIHSS score was 5 and KPS was 30 when admitted to hospital. Cranial MRI DWI revealed multiple acute infarctions on both cerebral hemispheres. (Fig. 4) The d-dimer was elevated to 8.23 mg/L. Antiplatelet treatment with aspirin and clopodorel was given but his neurological symptoms was not improved. Then elevated serum CEA (126 ng/L) and a tumor as well as cancerous lymphangitis in right lung on chest computed tomography was found. Tumor biopsy confimed the diagnosis of primary lung adenocarcinoma. (Fig. 1) Targeted therapy with icotinib (125 mg, tid) was administrated after EGFR 19 deletion was detected by tissue gene testing. One month later, he had significant improvement in mental status and aphasia. D-dimer and CEA decreased to normal level gradually. The primary lung tumor and cancerous lymphangitis was significantly shrank and a radiologically partial response was achieved (Fig. 5). His tumor was still under control and he has survived for 10 months.

Case 4 was a 58-year-old woman admitted with left-sided limb weakness that had persisted for 2 days. After admission, cranial imaging DWI suggested multiple infarct foci in the bilateral cerebral hemispheres. (Fig. 1) D-dimer was elevated to 28.9 mg/L and tumor marker CA125 was 116 U/ml. Chest CT revealed suspicious primary lung cancer with enlarged lymphnodes in mediastinum. Antiplatelet treatment were given but her condition worsened rapidly, with deterioration of mental drowsiness and NIHSS score of 14 points. She was diagnosed with primary lung adenocarcinoma after lung tumor biopsy, (Fig. 1) and genetic testing suggested EML4-ALK fusion mutaion. She received second-generation ALK-TKI enzatinib (200 mg qd). Her consciousness gradually improved after approximately one week of treatment. After one month, the patient's mental and physical condition improved significantly. D-dimer and CA125 decreased to normal level 3 months later. Chest CT showed decrease diameter in primary tumor, and a radiological PR was achieved and maintained till last follow up (Fig. 5).

## Discussion

Various causes of ischemic stroke, but malignant tumors as the origin is usually overlooked. In the present study, there were 10 patients diagnosed with lung adenocarcinoma with Trousseau syndrome. It is noteworthy that all 10 patients had stroke as the first symptom, and the lung malignancy lesion was found after examination; therefore, stroke can be included as one of the first manifestations in patients with undiagnosed lung tumor [8].

Trousseau syndrome is a clinically relevant coagulation abnormality in patients with cancer. In this study, we found that most patients with Trousseau syndrome had elevated serum D-dimer levels, which decreased after effective antitumor therapy. Therefore, if the patient is not diagnosed and treated at an early stage, it will lead to a persistent elevation of D-dimer levels, which will cause ischemic stroke. The data also illustrated that D-dimer levels can be used as a reference for the diagnosis and prognostic index of Trousseau syndrome in some cases. Nakamura et al. also found that D-dimer but not EGFR mutation status was a risk factor for overall survival [9]. It has been pointed out [10, 11] that the expression of tumor markers CEA and CA125 is associated with the occurrence of embolism in patients with cancer, and elevation of this index is associated with the recurrence of ischemic stroke of these patients. Most patients in this study had various degrees of elevated CEA and CA125; therefore, elevated tumor markers in patients with Trousseau syndrome were also considered to be associated with thrombosis.

All patients in this study started with neurological symptoms, including weakness of the extremities, dizziness, and slurred speech. The mean NIHSS score was 5.4 (range, 0–14), and the neurological symptoms caused by Trousseau syndrome were considered similar to those of general ischemic stroke. Imaging findings showed multiple infarct lesions on DWI in all patients. Therefore, additional attention is needed to screen for neoplastic lesions in patients with such features when performing imaging examinations.

The most effective treatment for Trousseau syndrome is antitumor therapy for the primary tumor [12], including targeted therapy, combined radiotherapy and surgery, and anticoagulation therapy. In this study, six out of ten patients received antitumor therapy; three of them showed significant improvement in symptoms and decrease in serum D-dimer levels and tumor markers after receiving antitumor therapy. Especially among 4 patients harbouring EGFR or ALK mutation, 3 patients achieved partial response and 1 stable disease, which is in accordance with the prospective study evaluating the efficacy of EGFR TKI for the treatment of lung cancer patients with poor performance status [13]. One patient died due to disease progression, four patients did not receive antitumor therapy but only anticoagulation, and two died due to disease progression.

In conclusion, we presented here the characteristic results of 10 NSCLC patients in this study with stroke as the first symptom. Patients with potential Trousseau syndrome together with the above characteristics should also be considered for the possibility of lung malignancy, and tumor-related tests should be clarified in a timely manner to achieve effective treatments. After diagnosing Trusso syndrome, the key to treatment is to combine antitumor therapy with anticoagulation therapy for the primary tumor, which is significantly more effective than anticoagulation therapy alone.

## Data Availability

All data generated or analysed during this study are included in this published article.
